# Integrating environment into One Health approaches: Embracing the complexity

**DOI:** 10.1016/j.onehlt.2025.101021

**Published:** 2025-03-26

**Authors:** Christina Lynggaard, Jan F. Gogarten, Pikka Jokelainen

**Affiliations:** a Section for Molecular Ecology & Evolution, Globe Institute, Faculty of Health and Medical Sciences, University of Copenhagen, Copenhagen, Denmark; b Helmholtz Institute for One Health Helmholtz-Centre for Infection Research (HZI) Department of Applied Zoology and Nature Conservation, University of Greifswald, Greifswald, Germany; c Infectious Disease Preparedness and One Health, Statens Serum Institut, Copenhagen, Denmark

**Keywords:** Biodiversity, Ecosystem, Environment, One Health

## Abstract

• Environmental surveillance provides important information, but is seldom considered.
• It is time to integrate the environment into One Health approaches.
• Advancements in methods and tools empower us to include complex environmental data.
• Embracing the complexity is best done together in multidisciplinary collaborations.

One Health is an integrated and unifying approach that recognizes that the health of humans, health of animals, health of plants and the shared environment are interconnected [1]. Many One Health initiatives, including research and pathogen surveillance activities, only touch upon part of the complexity of the approach, for example by focusing on a zoonotic pathogen that can infect both humans and other animals. The Quadripartite, comprising the Food and Agriculture Organization of the United Nations (FAO), the World Organisation for Animal Health (WOAH), the UN Environment Program (UNEP), and the World Health Organization (WHO), has highlighted the importance of integrating the broader environment into One Health initiatives, by including it as an Action Track in their One Health Joint Plan of Action [2].

The environment is shared by several organisms, including many different pathogens and a wide range of their potential host species. However, classically many studies have focused on a specific host species or on a particular pathogen. In reality, communities of hosts and their associated microorganisms vary across landscapes, with their interactions ultimately shaping transmission dynamics within and between species, and influencing disease emergence risk, e.g., through processes like the dilution effect, in which the presence of a diverse range of species reduces the encounters of a pathogen with efficient hosts and therefore reduces pathogen transmission. Data on the abiotic and biotic environment can provide insights into many processes shaping these communities, including changes in species interactions or transmission. Both biotic and abiotic factors are also often impacted to varying degrees of anthropogenic disturbance, affecting both the number and diversity of species found in a habitat, but also their behaviour, stress and ultimately susceptibility to infections. Importantly, the environment varies on both small and large spatial and temporal scales and depending on the factors being explored, different types and resolutions of environmental data will be informative.

In contrast to the synergistic vision of the One Health framework, the topics of human health, animal health, and environmental health are still often tackled in isolation [3]. Expanding pathogen surveillance approaches to include additional components, such as environmental factors and host communities, can complement established surveillance systems and provide new insights. For zoonotic disease surveillance, an integrated One Health approach can include data from testing samples from humans, animals, vectors and the environment, as applicable. Environmental surveillance, for example using wastewater samples, can provide complementary insights, e.g., on the extent of spread of pathogens, pollutants, and pharmaceutical residues [4]. Validating these new approaches in well-studied systems may allow such surveillance components to be rolled out in different settings. The environmental samples are more complex than the traditional clinical samples and a specific target is like a needle in the haystack. This complexity is also an asset, because the complex samples provide information on many different targets, thus generating rich data streams that may capture aspects of environmental complexity. It is time to embrace this diversity and complexity with the new methods and tools, both targeted and non-targeted, being developed to study it.

New tools are also improving our ability to effectively measure biodiversity in environmental samples. Among these, the analysis of DNA traces left by organisms in the environment, known as environmental DNA (eDNA), has been a valuable non-invasive and cost-effective tool for biodiversity surveys, particularly widely used in aquatic ecosystems. Environmental DNA has historically been mostly extracted from water, soil, and invertebrates, obtaining biodiversity data covering different organisms (e.g., mammals, birds, arthropods, bacteria) from the same sample. More recently, eDNA collected from novel sources such as air and deposited particles [5], which can be easily collected and processed, have revolutionized the use of eDNA for vertebrate monitoring in terrestrial ecosystems. The extent to which these tools could be used to monitor wildlife pathogens remains largely an open frontier, however with some examples such as use of flies to detect *Bacillus cereus* biovar *anthracis* [6], while they have already been integrated into prominent disease outbreak investigations (e.g., [7]). The ability to obtain fine-scale data on biodiversity opens an exciting possibility to generate datasets that can be linked to fine-scale data on human, animal or environmental health (e.g., personalized biodiversity data that can be linked to personalized medicine).

Similar to the ongoing advancements in molecular methods, the use of artificial intelligence has improved, e.g., acoustic monitoring and camera trap usage, enabling more precise and rapid species identifications. Importantly, different tools are often complementary revealing different components of biodiversity. Tools for measuring biotic (e.g., land use, land cover, plant productivity, plant diversity) and abiotic factors (e.g., snow and ice extent, temperature, humidity, precipitation, cloud cover, aerosol concentrations, greenhouse gases) with remote sensing technologies and networks of loggers are constantly improving, providing additional data layers on diverse factors at large spatial and temporal scales that enhance our understanding of biodiversity [8].

Although the complexity of ecosystems may seem daunting, integrating diverse data streams offers a unique opportunity to uncover possible intervention strategies that could mitigate the risk of disease emergence and spread. In fact, simplifying or ignoring these complexities risks generating misleading conclusions and insights and an overestimation of our ability to predict outcomes. Recent advances in diverse analytical tools—stemming from fields such as network sciences—provide a foundation for developing sophisticated models to capture intricate dynamics of ecosystems [9]. However, significant challenges remain in handling complex, interacting datasets, including issues of data integration, computational scalability, and the inherent uncertainty in modelling multifaceted ecological processes. Embracing the complexity and uncertainty is best done together in multidisciplinary collaborations that include biologists, ecologists and environmental scientists—fitting, as One Health aims to serve as a unifying approach.

## CRediT authorship contribution statement

**Christina Lynggaard:** Writing – review & editing, Writing – original draft, Resources, Conceptualization. **Jan F. Gogarten:** Writing – review & editing, Writing – original draft, Resources. **Pikka Jokelainen:** Writing – review & editing, Writing – original draft, Resources, Project administration, Conceptualization.

## Funding

PJ acknowledges funding from European Union's EU4Health programme under grant agreements 101102774 – EU-HIP, 101102733 – DURABLE, 101140460 – EU-WISH and 101132473 – OH4Surveillance. JFG acknowledges funding from the EU-Horizon Action Grant: “RESTOREID: Restoring ecosystems to stop the threat of re-emerging infectious diseases” and fruitful discussions with his colleagues from that consortium on this topic. CL was supported by a research grant from 10.13039/100008398VILLUM FONDEN (grant no. VIL41390). Views and opinions expressed do not necessarily reflect those of the European Union or HaDEA. Neither the European Union nor the granting authority can be held responsible for them.

## Declaration of competing interest

The authors declare no conflicts of interest.

## Data Availability

No data was used for the research described in the article.
